# Exploring the reasons for the sensitivity to change of a patient preference measure, compared with the KOOS questionnaire in patellofemoral osteoarthritis

**DOI:** 10.1186/1745-6215-16-S2-P66

**Published:** 2015-11-16

**Authors:** Matthew Parkes, Michael Callaghan, David Felson

**Affiliations:** 1 University of Manchester, Manchester, UK; 2 NIHR Manchester Musculoskeletal Biomedical Research Unit (BRU), Manchester, UK; 3 Boston University, Boston, MA, USA

## Background

We have previously found that one patient preference measure has increased sensitivity to change (Parkes et al. 2014, Arthritis Rheum 66 (11): S562). We explored the reasons why this particular measure has increased sensitivity, using data from the BRACE trial (ISRCTN50380458).

## Methods

The ‘pain on nominated aggravating activity’ visual analogue scale (VAS_NA_), was compared to the Knee Osteoarthritis Outcome Score (KOOS) questionnaire. The distribution of painful activities selected by patients using the VAS_NA_ in the BRACE trial was compared to activities covered by the KOOS. We also compared the standardised between-groups change in pain in the VAS_NA_ and the KOOS to see whether the magnitude in change was comparable.

## Results

Two-thirds of patients nominated pain using stairs, an activity captured by the KOOS. A further 28 subjects (22.2%) nominated squatting, bending or kneeling, and others nominated running (3.2%), activities not captured by KOOS. Standardised changes were generally higher for the VAS_NA_ than for the KOOS total score (for example, stairs subgroup change - 0.26 vs. -0.11), especially for patients choosing activities not covered by the KOOS (squatting, bending and kneeling subgroup change -0.26 vs. -0.16). In this subgroup, many of the individual KOOS items change little following treatment.

## Conclusion

The VAS_NA_ was more sensitive to change than the KOOS both in subgroups whose painful activity overlapped with KOOS items and those that did not. Questions which have less relevance may add ‘noise’ to the signal for change in pain, which could explain the KOOS's decreased sensitivity to change.

